# Increased Posterior Insula-Sensorimotor Connectivity Is Associated with Cognitive Function in Healthy Participants with Sleep Complaints

**DOI:** 10.3389/fnhum.2018.00035

**Published:** 2018-02-07

**Authors:** Chun-Hong Liu, Cun-Zhi Liu, Xue-Qi Zhu, Ji-Liang Fang, Shun-Li Lu, Li-Rong Tang, Chuan-Yue Wang, Qing-Quan Liu

**Affiliations:** ^1^Beijing Hospital of Traditional Chinese Medicine, Capital Medical University, Beijing Institute of Traditional Chinese Medicine, Beijing, China; ^2^Department of Radiology and Psychiatry, Beijing Anding Hospital, Capital Medical University, Beijing, China; ^3^The department of Acupuncture and Moxibustion, Beijing Hospital of Traditional Chinese Medicine, Capital Medical University, Beijing Key Laboratory of Acupuncture Neuromodulation, Beijing, China; ^4^Department of Psychosomatic Medicine/Administration of Medical Institution Conducting Clinical Trials for Human Used Drug, Beijing Hospital of Traditional Chinese Medicine, Capital Medical University, Beijing, China; ^5^Functional Brain Imaging Lab, Department of Radiology, Guang An Men Hospital, China Academy of Chinese Medical Sciences, Beijing, China

**Keywords:** resting-state, insular subregion, functional connectivity, insomnia, fMRI

## Abstract

Insomnia is characterized by sensory hypersensitivity and cognitive impairments. Recent work has identified the insula as a central brain region involved in both bottom-up gating of sensory information and top-down cognitive control. However, the specific relationships between insular subregion connectivity and emotional and cognitive functions remain unclear. In this study, resting-state functional magnetic resonance imaging data were obtained from 25 healthy participants with sleep complaints (HPS) and 25 age-, gender- and educational level-matched healthy participants without insomnia complaints (HP). We performed insular subregion (ventral anterior, dorsal anterior and posterior) functional connectivity (FC) analyses, and cognitive function was measured with several validated test procedures (e.g., the Wisconsin Card Sorting Test [WCST], Continuous Performance Test [CPT] and Trail making Test [TMT]). There were no significant differences between the two groups for WCST, CPT and TMT scores. The HPS group showed enhanced connectivity from the right posterior insula (R-PI) to the left postcentral gyrus (L-postCG) compared to HP group. WCST random errors (RE), sleep disturbance scores and HAMA scores correlated with this connectivity measurement in both HP and HPS groups. Our results provide direct evidence that the posterior insula (PI) synchronizes with sensorimotor areas to detect homeostatic changes and suggest that alteration of the latter is related to executive dysfunction in subjects with insomnia.

## Introduction

Insomnia and other sleep disorders are common clinical problems in primary health care and affect the health, safety and quality of life even in otherwise healthy individuals (Moore, 2012). Epidemiological studies indicate that 9%–15% of adults suffer from insomnia or another sleep disorder (Ohayon, 2002). It is widely recognized that sleep drives metabolite clearance from the brain (Xie et al., 2013) and transient perturbation during sleep can have a lasting impact on intrinsic brain activity (Buzsáki and Watson, 2012). In particular, insomnia has been associated with increased vulnerability to cardiovascular disease, psychiatric disorders, neurodegenerative disease, accidents and injuries (Wulff et al., 2010; Drummond et al., 2013). Insufficient sleep is also linked to impaired concentration, memory and cognitive impairments (Kyle et al., 2010; Fortier-Brochu et al., 2012). Drummond et al. (2013) demonstrated that subjects with primary insomnia have reduced right dorsolateral prefrontal cortex (dlPFC) modulation relative to good sleepers while performing an N-back working memory task. Pang et al. (2017) reported negative correlations between the functional connectivity (FC) strength within the default mode network (DMN) and Mini-Mental State Examination scores in chronic insomnia disorders. Wang et al. (2017) reported that the primary insomnia patients showed decreased connectivity between the left insula and a number of visual cognitive regions, including the right fusiform. Xu et al. (2016) discovered that deterioration of the throughput of a mathematical processing task was positively correlated with metabolism changes in the superior frontal gyrus in the context of sleep deprivation. Although sleep disturbance is associated with cognitive impairments, the potential relationship between its neurobiological mechanism and cognitive functions in healthy participants with insomnia complaints (HPS) remains clear.

A recent review has revealed that the neural correlates of insomnia symptoms are related to autonomic hyperarousal circuits (i.e., the hypothalamus and brainstem), emotional circuitry (i.e., the amygdala, hippocampus, and insula), and cognitive circuits (i.e., the dlPFC and ventrolateral prefrontal cortex [vlPFC]; Spiegelhalder et al., 2015). In particular, emotional circuit dysfunction might also contribute to the maintenance of insomnia symptoms (Baglioni et al., 2010). The insula is an important brain region within the emotional circuit and is associated with anxiety (Paulus and Stein, 2006). Abnormal insular activity and disrupted connectivity have also been noted in previous insomnia studies (Huang et al., 2012; Stoffers et al., 2012; Chen et al., 2014; Wang et al., 2016, 2017). For example, using simultaneous functional MRI (fMRI) and electroencephalography (EEG), Chen et al. (2014) reported increased coactivation of the anterior insula (AI) with the ventral AI (vAI) and dorsal AI (dAI) in insomniacs compared to healthy participants for the “fall asleep” scan, and AI activity is correlated with gamma frequency power. Using a delay discounting task, Martin et al. (2015) demonstrated decreased brain activation in the right inferior frontal gyrus, right middle frontal gyrus, and bilateral insula in obese adults with poor-quality sleep compared to obese adults with good-quality sleep. Using voxel-based morphometry, Stoffers et al. (2012) discovered that people with lower gray matter density in the left inferior orbitofrontal cortex (bordering the insula) had increased early morning awaking. Using resting-state FC of the amygdala, Huang et al. (2012) found decreased connectivity between the amygdale, insula, striatum and thalamus in patients with primary insomnia. Wang et al. (2016, 2017) performed resting-state FC analysis of the left insula and regional homogeneity (ReHo) analysis and found increased regional activity in the left insula and increased connectivity between the left insula and right anterior cingulate cortex (ACC) in patients with primary insomnia, whereas increased ReHo values of the left insula were positively correlated with anxiety scores. Interestingly, our previous study based on fractional amplitude of low frequency fluctuations (fALFF) analysis also revealed decreased fALFF in the left vAI and left posterior insula (PI) in healthy participants with insomnia symptoms (Liu et al., 2016). Existing structural and functional neuroimaging studies suggest that the insular cortex acts as an essential substrate for HPS (Carey et al., 2005); discrepancies across the above findings (increased vs. decreased insular density, activity, or connectivity) may be due to different statistical approaches or insular subregion functions.

Recent structural and FC studies suggest unique functions of the dAI, vAI and PI subregions, which are involved in cognition, affect processing and chemosensory function, and sensorimotor processing, respectively (Cauda et al., 2011; Deen et al., 2011; Chang et al., 2013). A number of neuroimaging findings indicate that the insula is involved in sensory and affective processing and high-level cognition (Paulus and Stein, 2010; Wei et al., 2016). Moreover, it is a central region of the salience network, which filters out irrelevant sensory signals from the body or environment and transmits salient information to support motor and cognitive function (Menon and Uddin, 2010). In the study by Wang et al. (2017) enhanced connectivity between the left insula and right ACC, right superior frontal gyrus, bilateral thalamus, and left precuneus, as well as decreased connectivity with the left middle temporal gyrus and right fusiform were revealed using left insula-based FC analysis that employed a combined height threshold *p* < 0.01 and a minimum cluster size of 18. The above research on primary insomnia has failed to fully resolve the relationship between the insular subregion-based FC changes and cognitive deficits. As abnormal connectivity with insula was mainly noted in emotional and cognitive-related regions in primary insomnia (Wang et al., 2017) and cognitive dysfunction in sleep disturbance has been suggested (Altena et al., 2008), research into insular subregional connectivity is particularly important. The aim of the current study was to investigate insular subregion-based FC patterns between HPS and healthy participants without sleep complaints (HP). We also examined the association between abnormal FC measurements and clinical scores (e.g., cognitive scores, sleep disturbance scores, adjusted Hamilton Depression Rating Scale [HAMD] and Hamilton Anxiety Rating Scale [HAMA] scores).

## Materials and Methods

### Subjects

Between 2009 and 2017, 25 adult HPS subjects were recruited from the local community using the Non-Patient Structured Clinical Interview for the Diagnostic and Statistical Manual of Mental Disorders (DSM-IV; SCID). Sleep complaints were assessed using three items on the HAMD that evaluated difficulty falling asleep, maintaining sleep or early awakening, and insomnia items on the HAMA (Hamilton, 1967). Participants who scored 1 or 2 on any three items from HAMD and scored 1 or 2 on HAMA insomnia items were considered to have sleep complaints. In addition, 25 age-, gender- and education-matched HP with good sleep quality and insomnia items who scored zero on both the HAMA and HAMD were also recruited from the local community. The criteria for healthy participants were as follows: (1) no history of dependence on alcohol or other substance abuse or other Axis I disorder; (2) 18–60 years of age; (3) right handed; and (4) medication naïve. We used the adjusted HAMD scores by omitted sleep items (e.g., 4 [insomnia-early], 5 [insomnia-middle], and 6 [insomnia-late]) to measure depression severity (Lowe et al., 2013), and the HAMA was used to measure anxiety severity. The current study was approved by the Institutional Review Board of the Beijing Anding Hospital, Capital Medical University, China. All participants provided written informed consent.

### Cognitive Measures

Executive function, sustained and selective attention, and attentive and executive functioning were assessed by the Wisconsin Card Sorting Test (WCST; Cavanna and Trimble, 2006), Continuous Performance Test (CPT; Altena et al., 2008), and Trailmaking Test (TMT; Groenewold et al., 2013), respectively. All these measures have been successfully used to detect cognitive impairment in individuals with insomnia (Wulff et al., 2010). The WCST comprises six categories and 48 cards and generates a number of psychometric scores, including total trials (TT), total correct (CT), total errors (TE), preservative errors (PE), random errors (RE) and categories. The CPT measures the number of correct detections of three tests and PE in CPT3. In CPT1, subjects were told to click the mouse whenever “4” was displayed within a series of numbers. In CPT2, subjects were told to respond whenever a “4” was displayed within eight numbers. In CPT3, subjects were told to respond whenever a “7” was displayed within eight numbers. The TMT includes two parts to test task completion time: TMT1 requires subjects to draw lines that connect consecutive numbers while still maintaining accuracy; TMT2 requires subject to alternately combine numbers with letters in ascending order while still maintaining accuracy.

### MRI Data Acquisition

Imaging data were collected on a Siemens TRIO 3.0T MRI scanner (Magnetom Verio, Siemens, Erlangen, Germany) in the National Key Laboratory for Cognitive Neuroscience and Learning, Beijing Normal University. The resting-state functional MRI (rsfMRI) acquisition parameters were acquired by a gradient echo-planar imaging (EPI) sequence as follows: time repetition (TR) = 2000 ms, time echo (TE) = 30 ms, flip angle (FA) = 90°, field of view (FOV) = 220 × 220 mm, repetition time matrix = 64 × 64, number of slices = 33, slice thickness = 3.5 mm, gap = 0.7 mm, and total 240 volumes in approximately 8 min. Participants were instructed to keep their eyes closed, think about nothing, and not fall asleep. High resolution three-dimensional T1-weighted sequence were acquired as follows: TR = 2530 ms, TE = 3.39 ms, slice thickness = 1.33 mm, gap = 0 mm, in-plane resolution = 256 × 256, FOV = 256 × 256 mm, inversion time (TI) = 1100 ms, and FA = 7° and 128 sagittal slices.

### Data Preprocessing

The rsfMRI data preprocessing steps were conducted using the Data Processing Assistant for Rest-State fMRI (DPARSF) toolbox^1^ in MATLAB (Chao-Gan and Yu-Feng, 2010). The first 10 time points were discarded for signal equilibrium, and the remaining 230 time points were then corrected for head movement. No participants had more than 2 mm translation or >2° in any angular dimension. Next, the remaining rsfMRI data were spatially normalized to the standard Montreal Neurological Institute (MNI) template using the EPI template and resliced at a resolution of 3 × 3 × 3 mm^3^. The obtained images were then smoothed with a 4-mm full width at half maximum (FWHM) Gaussian kernel. To minimize the effects of confounding factors and head motion to FC, six movement parameters and the averaged signals from white matter and cerebrospinal fluid were also removed as covariates. We did not regress out the global signal as suggested by Hahamy et al. (2014). We further calculated the mean frame-wise displacement (FD) to measure voxel-wise differences in motion in its derivation (Jenkinson et al., 2002), and the data were discarded when the mean FD was >0.2 (Jenkinson et al., 2002). At last, bandpass filtering (0.01–0.08 Hz) and linearly detrending were applied.

### FC Analysis

**Figure 1:**
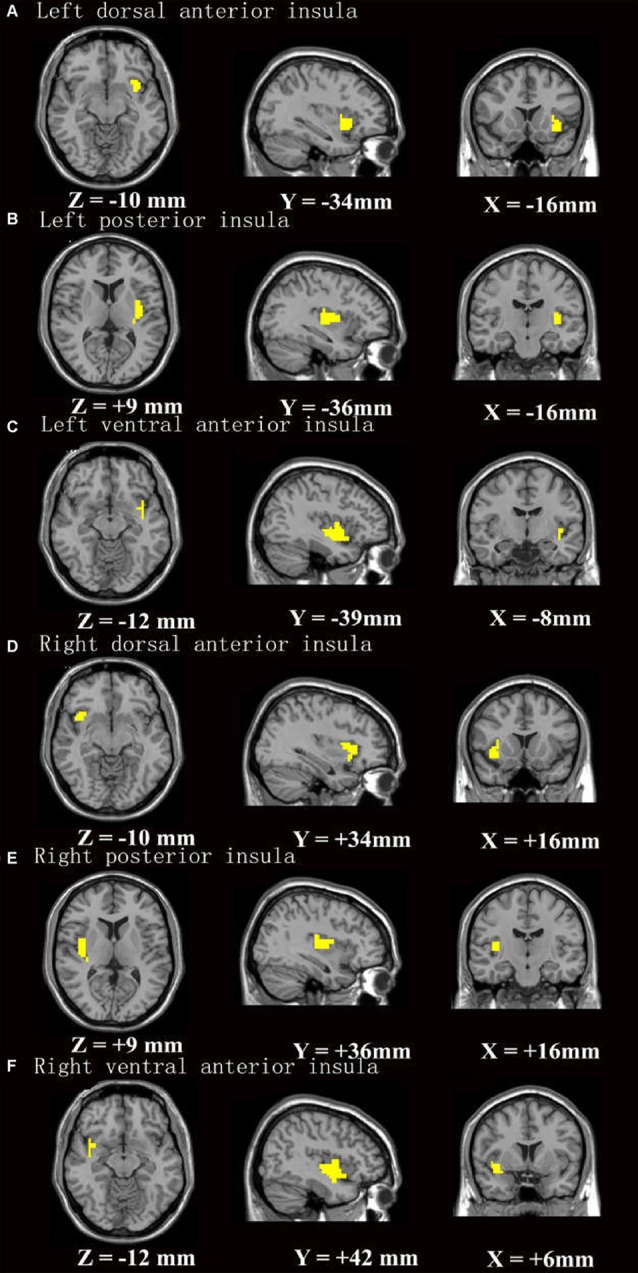
The insular subregion masks were based on Cauda et al. (2012). **(A–F)** Stands for insular subregion of left dorsal anterior insula, left posterior insula, left ventral anterior insula, right dorsal anterior insula, right posterior insula, right ventral anterior insula, respectively.

The FC analysis was conducted using the preprocessed data. The insular subregions masks created by Cauda et al. (2012; Figure 1) were identified as seed regions for further FC analysis (Song et al., 2011). Correlation coefficient maps were obtained by correlating the blood oxygenation level-dependent (BOLD) time course between the seed region and voxels within the whole brain. The correlation coefficients were converted to Fisher’s z-transformation to improve normality.

### Statistical Analysis

For clinical variables, group differences were independently assessed using two-sample, two-tailed *t*-tests or chi-squared tests (*p* < 0.05). Then, one-sample *t*-tests were performed for each group and then combined as a mask for subsequent two-sample *t*-testing. The significance level for each group was set at *p* < 0.001, and cluster size = 162 mm^3^ corrected for multiple comparisons. Two-sample *t*-testing was performed between the HPS and HP group with age, gender and educational level as covariates. The AlphaSim procedure^2^ was implemented in RESTplus, which was determined with updated Monte Carlo simulations in the AFNI AlphaSim program (Eklund et al., 2016). For example, the statistically corrected threshold of *p* < 0.05 was single voxel *p* = 0.001, FWHMx = 11.448 mm, FWHMy = 12.376 mm, FWHMz = 12.493 mm, cluster size = 891 mm^3^, and 1000 iterations for right posterior insula (R-PI) connectivity. Regions of interest (ROIs) were selected from averaging the time series of all voxels based on two-sample *t*-testing between the HPS and HP groups. Pearson correlation analyses were performed to investigate correlations between the mean Z scores in each ROI and the various clinical measures in all healthy participants. We also performed correlation analyses that excluded outliers, which were those with mean *z* scores +2 standard deviations (SDs; Geissler et al., 2007).

## Results

### Demographic and Clinical Data

As shown in Table 1, there were no significant differences between two groups with regard to age (*p* = 0.44), sex (*p* = 0.20), or educational level (*p* = 0.59). As expected, there were significant differences between the groups in sleep disturbance scores and adjusted HAMD and HAMA scores. However, the groups had similar scores for cognitive scales including the WCST, CPT and TMT. Please refer to Table 1 for more details. Mean FD did not differ between HPS (0.080 ± 0.032) and HP (0.077 ± 2480.033) in the final sample (*t*_(48)_ = 0.328, *p* = 0.744).

**Table 1:** Group demographics and clinical measures.

Measure (mean ± SD)	HPS (*N* = 25)	HP (*N* = 25)	Statistical value	*p* value
Sex (male/female)	15/10	14/11	1.28	0.20
Age (years)	37.12 ± 11.40	34.36 ± 13.43	0.78	0.44
Education level (years)	14.20 ± 3.57	14.72 ± 3.10	−0.55	0.59
Adjusted HAMD	1.24 ± 1.27	0.00 ± 0.00	4.89	0.00
HAMA	3.16 ± 1.89	0.28 ± 0.68	7.19	0.00
Sleep disturbance	1.68 ± 0.95	0.00 ± 0.00	8.89	0.00
CPT1	10.64 ± 0.86	10.72 ± 0.84	−0.33	0.74
CPT2	10.36 ± 1.85	10.08 ± 1.91	0.53	0.60
CPT3	10.68 ± 2.56	11.32 ± 2.23	−0.94	0.35
CPTPE	1.40 ± 2.31	1.00 ± 2.20	0.63	0.53
WCST TT	46.92 ± 2.22	47.32 ± 1.91	−0.68	0.50
CT	30.20 ± 7.70	29.12 ± 9.91	0.43	0.67
TE	16.72 ± 8.84	18.20 ± 10.58	−0.54	0.59
PE	12.12 ± 7.43	12.20 ± 7.99	−0.04	0.97
RE	4.60 ± 2.52	6.00 ± 4.58	−1.34	0.19
Categories	3.72 ± 1.79	4.00 ± 2.30	−0.48	0.63
TMT A	55.48 ± 11.51	54.00 ± 13.76	0.41	0.68
TMT B	84.16 ± 31.95	89.72 ± 36.38	−0.57	0.57

*Abbreviations: CPT, Continuous Performance Test; CT, number of correct trials; HAMA, Hamilton Anxiety Rating Scale; HAMD, Hamilton Depression Rating Scale; HP, healthy participants without sleep complaints; HPS, Healthy participants with sleep complaints; PE, preservative errors; RE, random errors; SD, standard deviation; TE, total number of errors; TMT, Trail-making Test; TT, number of total trials*.

### Insular Subregion Seed-Based FC Analyses

**Figure 2:**
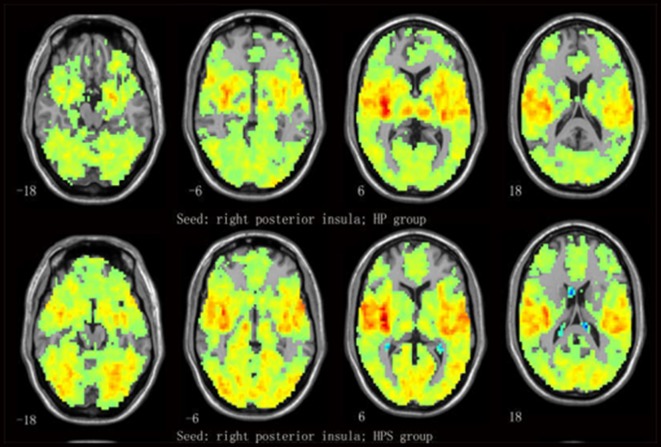
Mean functional connectivity (FC) strength maps for the healthy participants with sleep complaints (HPS) and healthy participants without sleep complaints (HP) groups. One-sample *t*-tests on individual FC values were conducted at each voxel using the right posterior insula (R-PI) as a seed.

One-sample *t*-tests revealed that the insular subregion had extensive FC with the frontal, temporal, parietal, occipital and cerebellar regions in both groups (Figure 2 and Supplementary Figure S1).

### Group Differences in FC

**Figure 3:**
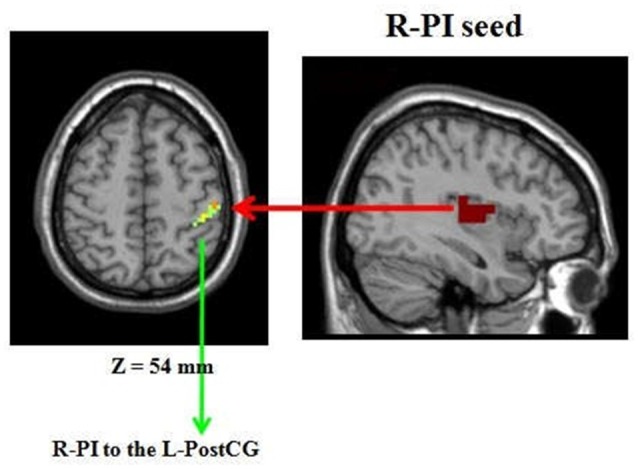
Comparison of healthy participants with sleep complaints (HPS) and healthy participants without sleep complaints (HP) groups. Clusters representing greater connectivity from the R-PI seed to the left postcentral gyrus (L-postCG) with the strict AlphaSim correction of significant thresholds of *p* < 0.001.

Compared to the HP group, the right PI (R-PI)-based FC analysis showed increased FC with the left postcentral gyrus (L-postCG) in HPS subjects (Figure 3 and Table 2). To obtain a measure of effect size, we calculated Cohen’s *d* values for the mean Z scores within the L-postCG cluster. The mean L-postCG Z scores values were 0.38 (SD 0.14) and 0.21 (SD 0.13) for the HPS and HP groups, respectively. The corresponding Cohen’s *d* of 1.26 can be conventionally interpreted as constituting a large effect size.

**Table 2:** Comparisons of insular subregion seed-based functional connectivity between healthy participants with insomnia complaints and healthy participants without insomnia complaints.

	Brain regions	Side	Brodmann areas	MNI coordinates			*K*	*t* statistic
				*x*	*y*	*z*		
*p* < 0.001								
R-PI seed	Postcentral gyrus	Left		−51	−24	57	36	5.81

*Abbreviations: K, cluster number; MNI, Montreal Neurological Institute; R-PI, right posterior insula*.

### Correlations between FC and Clinical Data in all Healthy Participants

**Figure 4:**
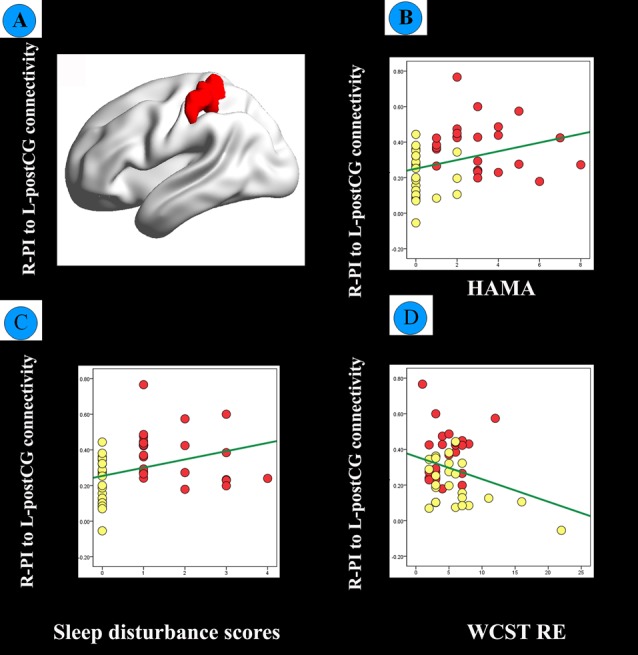
**(A)** FC between the R-PI and L-postCG was significantly different between healthy participants with sleep complaints (HPS, red dots) and healthy participants without sleep complaints (HP, yellow dots) based on a two-sample *t*-test controlled for age, gender, educational level. **(B)** Scatter plot of the correlation between R-PI- L-postCG connectivity and the Hamilton Anxiety Rating Scale (HAMA) score. **(C)** Scatter plot of the correlation between the R-PI- L-postCG connectivity and sleep disturbance scores. **(D)** The scatter plot of the correlation between R-PI- L-postCG connectivity and Wisconsin Card Sorting Test random errors (WCST RE) scores.

Sleep disturbance and HAMA scores correlated with R-PI to L-postCG connectivity in all healthy participants. Moreover, RE in the WCST negatively correlated with R-PI to L-postCG connectivity in all healthy participants (Figure 4). The detailed correlation results between FC and the various clinical measures in all healthy participants are presented in Table 3. Correlation analyses that excluded the outliers yielded similar results (Supplementary Table S1).

**Table 3:** Correlation between brain areas with significant between-group functional connectivity difference and various clinical measures.

Brain regions	R-PI to L-postCG
CPT 1	0.26
CPT 2	0.27
CPT 3	0.03
CPT PE	−0.04
WCST TT	−0.20
CT	0.20
TE	−0.22
PE	−0.13
RE	−0.30*
Categories	0.10
TMT A	−0.17
TMT B	−0.19
Sleep disturbance scores	0.32*
Adjusted HAMD	0.18
HAMA	0.31*

Abbreviations: CPT, Continuous Performance Test; PE, preservative errors; TT, number of total trials; CT, number of correct trials; TE, total number of errors; RE, random errors; TMT, Trail-making Test; HAMD, Hamilton Depression Rating Scale; HAMA, Hamilton Anxiety Rating Scale; R-PI, right posterior insula; L-PostCG, left postcentral gyrus; *stands for *p* < 0.05.

## Discussion

By comparing the FC of insular subregions in individuals with and without sleep complaints, we found that those with HPS showed greater FC of the R-PI with L-postCG. Our results emphasize a distinctive function of PI in that it synchronizes with sensorimotor areas to sense homeostatic changes. Moreover, there were relationships between R-PI to sensorimotor connectivity and cognitive function and sleep disturbance scores in all healthy participants, suggesting that the PI synchronizes with areas related to executive dysfunction.

The insular cortex, or “Island of Reil”, has gained great interest since the development of functional neuroimaging techniques (Uddin et al., 2017). Most studies (Cauda et al., 2011; Deen et al., 2011; Chang et al., 2013) have divided the insular cortex into three functionally distinct regions: the vAI, which has strong connections with the limbic regions (e.g., the amygdala and ventral striatum) and is involved in affective salience; the dAI, which has strong connections with the frontal operculum and ACC and participates in higher-level cognitive processing such as motivation and decision making; and the PI, which has strong connections with sensorimotor areas and monitors pain and interoceptive processing (Strigo and Craig, 2016). Our one-sample *t*-test results confirmed that the vAI synchronized with emotional regions, the dAI synchronized with cognitive regions and the PI synchronized with sensorimotor areas (see Figure 1 and Supplementary Figure S1 for details). Our finding of comparable cognitive function between the HPS and HP groups is well aligned with the lack of group differences in FC of the vAI or dAI with other brain regions involved in cognitive and emotion processing. These results suggest that sleep complaints might be a prodromal stage of insomnia and therefore differ from insomnia with regard to cognitive symptoms and neural substrates related to cognitive and emotional processing. With respect to lateralization, the left insula is predominantly associated with parasympathetic activity, positive emotional stimuli and “energy enrichment” whereas the right insula is associated predominantly with sympathetic activity, negative emotional stimuli, and “energy expenditure” (Strigo and Craig, 2016). The increased R-PI-sensorimotor cortex FC in the HPS group and its correlation with insomnia symptoms might indicate that altered bottom-up processing is the most prominent deficit in the HPS group.

The major goal of the current study was to investigate the relationship between cognitive function and insular subregion-based FC abnormalities. Cognitive dysfunction in insomnia is commonly reported (Kyle et al., 2010; Fortier-Brochu et al., 2012). A meta-analysis indicated that individuals with insomnia exhibit small-to-moderate impairments on tasks assessing episodic memory, problem solving, and working memory (Fortier-Brochu et al., 2012). However, the present study did not find any group differences in cognitive scales between the HPS and HP groups. This might be due to the heterogeneity of research samples since all of the healthy participants had sleep complaints but not insomnia. However, we found that R-PI to L-postCG connectivity was positively associated with HAMA scores and sleep disturbance scores, while it was negatively associated with WCST RE scores. These findings are consistent with a hyperarousal state, which is associated with high sensitivity and cognitive dysfunction while falling asleep (Riemann et al., 2010; Killgore, 2013). Moreover, we found increased R-PI to L-postCG FC in the HPS group. Similarly, compared with HP, chronic primary insomnia patients have increased connectivity (Li et al., 2017) and local intrinsic fluctuations in the somatosensory cortex during the resting state (Zhou et al., 2016b). Killgore (2013) found that latency to fall asleep was associated with augmented connectivity between the primary visual cortex and supplementary motor cortex, which suggests sustained sensory processing of environmental stimuli. Moreover, Zhou et al. (2016a) reported hyperconnectivity in the sensorimotor network in subjects with chronic primary insomnia, indicating that prolonged sleep onset might be due to the hyperarousal model in sensory processing (Huang et al., 2012). According to these studies, it seems plausible to postulate that worsening sleep quality is often comorbid with anxiety (Ohayon and Roth, 2003; Riemann et al., 2010). In addition, increased insula-sensorimotor connectivity was associated with cognitive dysfunction (e.g., WCST RE and CPT2 scores). The detrimental effect of insufficient sleep on cognitive dysfunction is well established (Killgore, 2013), but further work is required to clarify the causal relationship between insula-somatosensory connectivity and cognitive dysfunction.

In this study, we adopted a previous parcellation result to investigate insular subregion-based FC changes. Cauda et al. (2012) parcellated the insula on the basis of meta-analytic connectivity modeling and meta-analytic clustering, while Deen et al. (2011) considered the basis of resting state clustering patterns. On the other hand, Cauda et al. (2012) used the BrainMap database including 22,872 healthy participants and a total of 2957 foci from 1305 articles involved in active tasks, while Deen et al. (2011) used a resting-state FC clustering technique to assess 30 healthy participants wit resting-state scans and 20 healthy participants completed a disgusting/neutral image experiment (four of whom had also received resting scans). The insula parcellation results reported by Cauda et al. (2012) are similar to those described by Deen et al. (2011).

Some limitations should be mentioned regarding our study. First, the sample size was relatively small. Second, additional neuropsychological and cognitive tests (e.g., the Pittsburgh sleep quality test, Duke structured interview and Stroop Color Word Test; Howieson et al., 2004) are also needed. Third, the HAMA also includes some sleep factors, and the correlation between altered connectivity patterns and HAMA score may also be influenced by anxiety. Finally, fMRI data on the cognitive tasks are needed to verify our current findings.

## Conclusion

Our results suggest that healthy participants with insomnia complaints have increased insular connectivity with sensorimotor brain regions. This is direct evidence that the PI synchronizes with sensorimotor areas to sense homeostatic changes. Specifically, we observed an association between R-PI to sensorimotor connectivity and cognitive function and sleep disturbance scores in both HP and HPS groups. These findings indicate that increased insula-sensorimotor connectivity is a core deficit of disordered sleep, and individuals with decreased cognitive function might be prone to insomnia.

## Author Contributions

C-HL and C-ZL designed the study, along with J-LF, C-YW and Q-QL. C-HL, C-ZL, X-QZ, J-LF, S-LL, L-RT and C-YW collected the original imaging data. C-HL, J-LF and C-YW managed and analyzed the imaging data. C-HL, C-ZL, X-QZ and J-LF wrote the manuscript. All authors contributed to and have approved the final manuscript.

## Conflict of Interest Statement

The authors declare that the research was conducted in the absence of any commercial or financial relationships that could be construed as a potential conflict of interest.

## Footnotes

^1^http://rfmri.org/DPARSF

^2^https://afni.nimh.nih.gov/pub/dist/doc/manual/AlphaSim.pdf
