# Supplementary material for: Increased Posterior Insula-Sensorimotor Connectivity Is Associated with Cognitive Function in Healthy Participants with Sleep Complaints
Source: Front Hum Neurosci. 2018 Feb 7;12:35. doi: 10.3389/fnhum.2018.00035 (PMC5808346; doi:10.3389/fnhum.2018.00035)
Supplement: Supplementary file 1 [file Image_1.pdf]

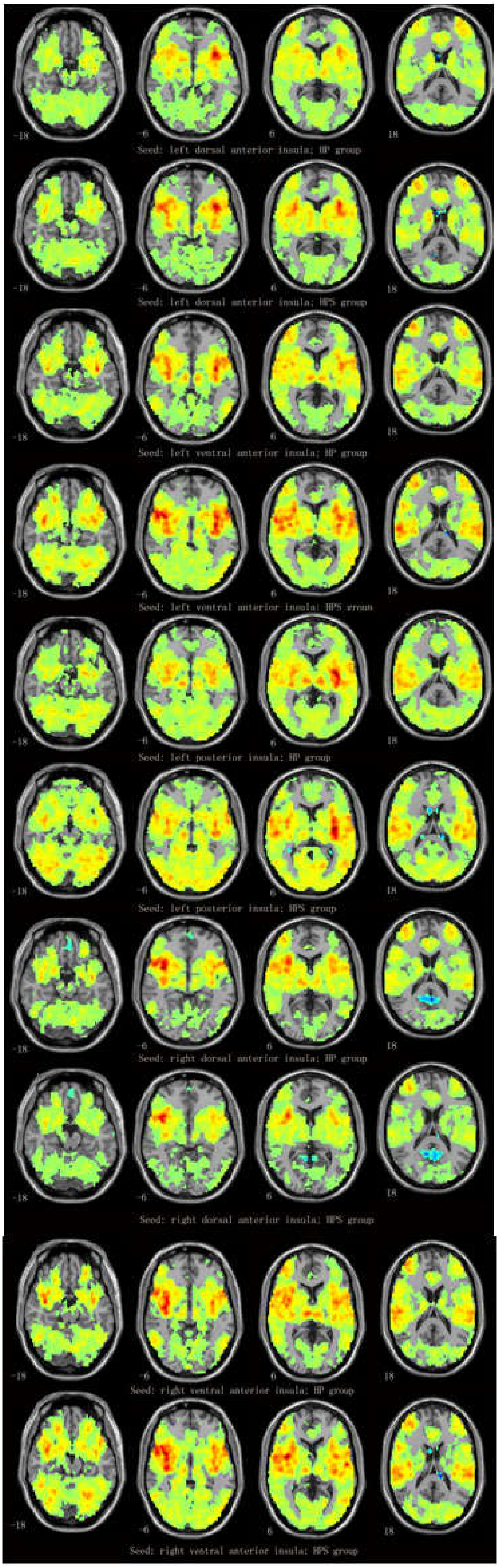

-18
-6
6
18
Seed: left dorsal anterior insula; HP group
-18
-6
6
18
Seed: left dorsal anterior insula; HPS group
-18
-6
6
18
Seed: left ventral anterior insula; HP group
-18
-6
6
18
Seed: left ventral anterior insula; HPS group
-18
-6
6
18
Seed: left posterior insula; HP group
-18
-6
6
18
Seed: left posterior insula; HPS group
-18
-6
6
18
Seed: right dorsal anterior insula; HP group
-18
-6
6
18
Seed: right dorsal anterior insula; HPS group
-18
-6
6
18
Seed: right ventral anterior insula; HP group
-18
-6
6
18
Seed: right ventral anterior insula; HPS group

Supplement Figure 1. Mean functional connectivity strength maps within the healthy participants with sleep complaints (HPS) or healthy participants without sleep complaints (HP) groups. One-sample *t*-tests on the individual functional connectivity values were conducted at each voxel with left ventral anterior insula, left dorsal anterior insula, left posterior insula, and right dorsal anterior insula as seed.

Supplement Table 1. Correlation between R-PI to L-postCG connectivity and various clinical measures.

Brain regions	R-PI to L-postCG
CPT2	0.20
WCST RE	-0.32*
sleep disturbance scores	0.34*
HAMA	0.39*

Abbreviations: CPT: Continuous Performance Test; L-PostCG, left postcentral gyrus; RE: random errors; R-PI, right posterior insula; R-PreCG, right precentral gyrus. The values in the table are Person's Correlation Coefficients; * stands for $p < 0.05$.
